# 

**Published:** 1890-07-12

**Authors:** 


### NOTICE TO CORRESPONDENTS.

All MS., Letters, Books for Review, and other matters intended for the Editor should be addressed The Editor, The Lodge, Porchester Square, London, W.

The Editor cannot undertake to return rejected M.S., even when accompanied by stamped directed envelope.

### Notice to Advertisers and Subscribers.

All Advertisements, Orders for Copies of The Hospital, and Business Communications should be addressed to The Publisher, *not to the Editor*, The Hospital, 140, Strand, W.C.

### Bound Volumes of "The Hospital."

Vol. VI., containing full page portraits of T.R.H. the Prince and Princess of Wales, and Miss Florence Nightingale, is obtainable at 4s., post free.

Orders will be received for Vol. VII., 4s. post free. Cases for binding may be had of the Publisher, at 1s. 6d. each.

### To Residents, Secretaries, and Matrons.

The Editor will be glad to have the Regulations and each Report as issued by Asylums, Hospitals, Convalescent and Nursing Institutions, as well as those of other Charities, and an early copy in duplicate of all Appeals and Festival Notices, etc., addressed to *The Editor, The Lodge, Porchester Square, London, W.* Any superintendent, secretary, matron, or other chief official who regularly sends such information may be registered, on application to the Publisher, to receive free of cost a cover for binding The Hospital in half-yearly volumes.

### BURDETT'S HOSPITAL ANNUAL FOR 1890

Is now ready, and may be obtained of the Publisher, The Hospital Offices, 140, Strand, W.C., price 2s. 6d. limp boards, or 3s. 6d. cloth. All orders must be accompanied by a remittance.

### Scraps and Gleanings.

Warwick will keep Hospital Saturday on September 6th.

Four people have been poisoned at Monkstown by eating mussels.

Miss Eleanor Fleury has just been made a Bachelor of Medicine of Dublin University.

Miss Blanche Thompson passed the Apothecaries' Hall (Assistants) Examination on June 25th.

Doctor Brouardel, of Paris, says that since 1877 the number of madmen has increased threefold. He believes this is due to drink.

Miss Weston's work among the sailors continues to prosper. During the past year 3,336 pledges were taken at Devonport and Portsmouth.

The trustees of the Smith Charity have sent Mr. Lyttelton £100 for the Children's Country Holidays Fund (10, Buckingham Street, Strand).

Miss E. F. Foster has founded a scholarship of £30 per annum at Dundee University College, to promote original research in the chemical department.

An earnest appeal is made on behalf of the new Home for Destitute Boys in Shaftesbury Avenue, to discharge a debt of £3,300 remaining on the building.

Under the patronage of Princess Mary, Duchess of Teck, a morning concert will be given on the 14th of this month at the residence of Lady McKenna, in aid of the Home of Rest for Horses.

Several deaths occur daily from small-pox at Assouan, and many fatal cases of typhus are reported from Wady Halfa. Preparations are being made at the latter place for the reception of troops.

Princess Mary of Teck and her daughter the Princess Victoria were present at the matinée at the Criterion Theatre in aid of the poor of St. Luke's, Camberwell, S.E. The gross receipts were close on £400.

The drawing-room meeting at Mrs. W. H. Smith's on June 27th, in aid of the Children's Country Holidays Fund (10, Buckingham-street, Strand), proved a very great success. A sum of over £270 was subscribed.

Mr. J. Geare, of Exeter, who has already made numerous donations to the Devon and Exeter Hospital, has added to the number by placing £1,400 in the 2½ per cents. at the disposal of the Samaritan Fund of the hospital.

Dr. Rentoul has given a resumé of the laws and regulations relating to midwives abroad, together with his views on the Midwives Bill now before Parliament, in a paper published in the *Provincial Medical Journal* of July.

Sir John Lubbock and other gentlemen have presented a petition to the Queen in Council, praying for the grant of a Charter of Incorporation as the President and Governors of the Royal London Ophthalmic Hospital.

### Books Received.

We have recently received the following books for review:

Adlard and Son.
"Papers on Surgery, Pathology, and Allied Subjects." F. Le Gros Clark, F.R.S.

Bale and Sons.
"Nasal Obstruction and Mouth Breathing." Spicer.

Churchill, J. and A.

"Tropical Climates and Indian Diseases." Sir W. Moore, K.C.I.E.

Davis, F. A.

"A System of Practical and Scientific Physiognomy." Mary Olmsted Stanton.

"Practical Electricity in Medicine and Surgery." G. A. Liebig, Jr., Ph.D., and George H. Rohé, M.D.

Keener, W. T. (Chicago).

"Rectal and Anal Surgery." Edmund Andrews, M.D., LL.D., and E. W. Andrews, A.M., M.D.

"Dental Chemistry and Metallurgy" (2nd edition). C. Mitchell, M.D.

"Intestinal Surgery." N. Senn, M.D., Ph.D.

Lewis, H. K.

"Massage and the Swedish Movements." K. W. Ostrom.

Livingstone, E. and S.
"Nerves of the Human Body." A. W. Hughes.

Low, Sampson, and Co.
"Antiseptics in Surgery." E. S. Bishop.

Nisbet and Co.

"What Cheer O?" Alexander Gordon.

Renshaw and Co.
"Forensic Medicine." Armand Semple.

Swan, Sonnenschein, and Co.
"Bismarck and State Socialism." W. H. Dawson.
"Godwin's 'Political Justice.'" Edited by H. S. Salt.
"Mad Doctors." By One of Them.

Ward and Lock.
"London Medical Specialists." C. B. Allen.

Washington Bureau of Information.

"English-Eskimo and Eskimo-English Vocabularies." Wells and Kelly.

"History of Federal and State Aid to Higher Education in the United States." Blackmar.

"Proceedings of the Department of Superintendence of the National Educational Association at its Meeting at Washington, 1889."

